# Supplementary material for: Evaluating an Early Risk Model for Uncomplicated Hypertension in Pregnancy Based on Nighttime Blood Pressure, Uric Acid, and Angiogenesis-Related Factors
Source: Int J Mol Sci. 2025 Jun 25;26(13):6115. doi: 10.3390/ijms26136115 (PMC12249693; doi:10.3390/ijms26136115)
Supplement: Supplementary file 1 [file ijms-26-06115-s001.zip › Supplementary Table S1.pdf]

Supplementary Table S1. Twenty-four-hour ambulatory blood pressure monitoring indices. Comparison between the groups.

Variable	Total (n=132)	UH[a] (no) (n= 60)	UH (yes) (n= 72)	p-value
24-hSBP (mmHg)	122.0 (18.0)	118.0 (9.8)	130.5 (18.5)	<0.001
dSBP (mmHg)	127.0 (15.0)	122.0 (12.0)	132.5 (17.5)	<0.001
nSBP (mmHg)	115.0 (23.5)	107.0 (13.8)	125.5 (18.8)	<0.001
nSBP dipping (%)	8.1 (9.4)	12.4 (8.6)	5.9 (7.7)	<0.001
24-hDBP (mmHg)	73.0 (11.8)	71.5 (6.8)	77.5 (16.0)	<0.001
dDBP (mmHg)	77.0 (11.8)	75.5 (8.0)	81.8 (12.0)	<0.001
nDBP (mmHg)	66.5 (16.0)	62.0 (10.0)	75.0 (15.8)	<0.001
nDBP dipping (%)	12.3 (10.3)	15.6 (12.3)	9.9 (9.1)	<0.001
24-hHR (bpm)	83.0 (12.5)	81.5 (13.8)	83.0 (11.5)	0.042
dHR (bpm)	85.0 (13.0)	85.0 (15.8)	86.0 (11.8)	0.060
nHR (bpm)	77.0 (11.0)	75.0 (12.5)	77.0 (9.0)	0.017

[a] Patient groups according to the presence of an uncomplicated hypertension at the 20th week of pregnancy. Results expressed as † refer to the median and interquartile range. ABPM—Ambulatory blood pressure monitoring; BP—Blood pressure; SBP—Systolic BP; 24-hSBP—24-hour SBP; dSBP—Daytime SBP; nSBP—Nighttime SBP; DBP—Diastolic BP; 24-hDBP—24-hour DBP; dDBP—Daytime DBP; nDBP—Nighttime DBP; HR—Heart rate; 24-hHR—24-hour HR; dHR—Daytime HR; nHR— Nighttime HR; ; HbA1c—Glycated hemoglobin mmHg—Millimeter of mercury; %—Percentage; bpm—Beats per minute.
