# Supplementary material for: Evaluating an Early Risk Model for Uncomplicated Hypertension in Pregnancy Based on Nighttime Blood Pressure, Uric Acid, and Angiogenesis-Related Factors
Source: Int J Mol Sci. 2025 Jun 25;26(13):6115. doi: 10.3390/ijms26136115 (PMC12249693; doi:10.3390/ijms26136115)
Supplement: Supplementary file 1 [file ijms-26-06115-s001.zip › Supplementary Table S2.pdf]

Supplementary Table S2. Logistic regression models based on 24-hour ABPM indices and the UA-AF Index for predicting UH during pregnancy: 24-h and daytime BP indices

Variable	B	SE	Wald	p-value	Exp(B)	95%CI (lower)	95%CI (upper)
24-hSBP (accuracy: 77%. Nagelkerke R^2: 0.331)							
Former smoker	1.410	0.707	3.977	0.046	4.097	1.025	16.383
HT drugs (yes)	1.257	0.626	4.034	0.045	3.516	1.031	11.989
24-hSBP (mmHg)	0.080	0.020	16.689	<0.001	1.083	1.042	1.125
Constant	–10.023	2.405	17.372	<0.001			
24-hSBP + UA-AF index (accuracy: 75%. Nagelkerke R^2: 0.447)							
Former smoker	1.696	0.784	4.680	0.031	5.451	1.173	25.337
HT drugs (yes)	1.257	0.663	3.601	0.058	3.516	.960	12.882
24-hSBP (mmHg)	0.076	0.020	14.097	<0.001	1.079	1.037	1.123
UA-AF index (SU)	0.979	0.309	10.009	0.002	2.661	1.451	4.880
Constant	–9.852	2.518	15.311	<0.001			
Daytime SBP (accuracy: 74%. Nagelkerke R^2: 0.289)							
Former smoker	1.503	.694	4.685	0.030	4.495	1.153	17.530

HT drugs (yes)	1.250	.617	4.110	0.043	3.490	1.042	11.688
dSBP (mmHg)	0.069	.019	13.161	<0.001	1.072	1.032	1.113
Constant	–8.997	2.426	13.755	<0.001			
Daytime SBP + UA-AF index (accuracy: 75%. Nagelkerke R^2: 0.436)							
Former smoker	1.790	0.767	5.445	0.020	5.990	1.332	26.942
HT drugs (yes)	1.302	0.661	3.882	0.049	3.675	1.007	13.413
dSBP (mmHg)	0.064	0.020	10.611	0.001	1.066	1.026	1.109
UA-AF index (SU)	0.965	0.300	10.329	0.001	2.626	1.457	4.730
Constant	–8.587	2.515	11.657	0.001			
24-hDBP (accuracy: 75%. Nagelkerke R^2: 0.315)							
Former smoker	1.661	.706	5.532	0.019	5.265	1.319	21.013
HT drugs (yes)	1.505	.622	5.863	0.015	4.505	1.332	15.235
24-hDBP (mmHg)	0.105	.027	15.173	<0.001	1.110	1.053	1.171
Constant	–8.044	2.013	15.970	<0.001			
24-hDBP + UA-AF index (accuracy: 75%. Nagelkerke R^2: 0.446)							
Former smoker	1.956	0.819	5.698	0.017	7.071	1.419	35.233

HT drugs (yes)	1.507	0.664	5.159		0.023	4.514	1.229	16.572
24-hDBP (mmHg)	0.103	0.028	13.574		<0.001	1.108	1.049	1.170
UA-AF index (SU)	0.976	0.309	9.950		0.002	2.653	1.447	4.864
Constant	–8.078	2.082	15.055		<0.001			
Daytime DBP (accuracy: 73%. Nagelkerke R^2: 0.291)								
Former smoker	1.702	.698	5.952		0.015	5.485	1.397	21.532
HT drugs (yes)	1.463	.613	5.689		0.017	4.317	1.298	14.359
dDBP (mmHg)	0.094	.026	13.049		<0.001	1.098	1.044	1.156
Constant	–7.521	2.027	13.761		<0.001			
Daytime DBP + UA-AF index (accuracy: 73%. Nagelkerke R^2: 0.431)								
Former smoker	2.002	0.808	6.142	1	0.013	7.402	1.520	36.047
HT drugs (yes)	1.474	0.661	4.983	1	0.026	4.368	1.197	15.942
dDBP (mmHg)	0.093	0.027	12.211	1	<0.001	1.097	1.042	1.156
UA-AF index (SU)	1.004	0.309	10.548	1	0.001	2.730	1.489	5.005
Constant	–7.649	2.074	13.603	1	<0.001			

Logistic regression models elucidating the relationship of BP indices and UA-AF index (predictor variables) with UH (no/yes). All models incorporated data from 132 pregnant women. ensuring no missing data. The variables to be controlled were BMI. HT drugs. essential HT and former smoker. For all the models: Omnibus test of model coefficients (p-value) < 0.05; Hosmer-Lemeshow test (p-value)> 0.05. ABPM—Twenty-four-hour ambulatory blood pressure monitoring; UH—Uncomplicated hypertension; HT—Hypertension; UA—Uric acid; AF—Angiogenesis-related factors; UA-AF Index—Uric acid and angiogenesis-related factors ratio Index; BP—Blood pressure; SBP—Systolic BP; 24-hSBP—24-hour SBP; dSBP—Daytime SBP; DBP—Diastolic BP; 24-hDBP—24-hour DBP; dDBP—Daytime DBP; mmHg—Millimeter of mercury; %—Percentage; SU—Standard unit.
